# Nutraceutical and Probiotic Approaches to Examine Molecular Interactions of the Amyloid Precursor Protein APP in *Drosophila* Models of Alzheimer’s Disease

**DOI:** 10.3390/ijms22137022

**Published:** 2021-06-29

**Authors:** David Jalali, Justine Anne Guevarra, Luz Martinez, Lily Hung, Fernando J Vonhoff

**Affiliations:** 1 Department of Biological Sciences, University of Maryland Baltimore County, Baltimore, MD 21250, USA; ejal1@umbc.edu (D.J.); luzmar1@umbc.edu (L.M.); lilyh1@umbc.edu (L.H.); 2 Department of Biological Sciences, Towson University, Towson, MD 21252, USA; jgueva2@students.towson.edu

**Keywords:** nutrition, neurodegeneration, gut microbiome, insect disease models

## Abstract

Studies using animal models have shed light into the molecular and cellular basis for the neuropathology observed in patients with Alzheimer’s disease (AD). In particular, the role of the amyloid precursor protein (APP) plays a crucial role in the formation of senile plaques and aging-dependent degeneration. Here, we focus our review on recent findings using the *Drosophila* AD model to expand our understanding of APP molecular function and interactions, including insights gained from the fly homolog APP-like (APPL). Finally, as there is still no cure for AD, we review some approaches that have shown promising results in ameliorating AD-associated phenotypes, with special attention on the use of nutraceuticals and their molecular effects, as well as interactions with the gut microbiome. Overall, the phenomena described here are of fundamental significance for understanding network development and degeneration. Given the highly conserved nature of fundamental signaling pathways, the insight gained from animal models such as *Drosophila melanogaster* will likely advance the understanding of the mammalian brain, and thus be relevant to human health.

## 1. Introduction

Alzheimer’s disease (AD) is one of the most well-known and widespread neurodegenerative diseases worldwide [1,2]. It is estimated that around 50 million people live with dementia and that 60–70% of these people live with AD, with a projected increase to 152 million by 2050 [3,4]. Thus, it is of paramount importance to explore novel avenues of research which may help in treating the disease, slowing its progress, and potentially even preventing its onset altogether. Although there is still no cure for Alzheimer’s disease, it is worth highlighting some novel treatments that would require further testing based on promising initial results. We will mostly discuss nutraceutical compounds (naturally occurring chemicals in food that may have medicinal benefits) as well as synbiotic formulations and their interactions with the gut microbiome tested in *Drosophila* that have the potential to alleviate AD-related symptoms. In addition, we will highlight recent studies using *Drosophila* AD models that have expanded our understanding of the molecular mechanisms underlying AD-associated phenotypes, with a specific focus on the amyloid precursor protein (APP) and its fly homolog APP-like (APPL).

## 2. AD Symptoms, Progression and Diagnosis of Neuropathology

Key symptoms of AD mainly involve declining levels of cognition, specifically through loss of short and long-term memory [5]. In addition, patients can develop problems with their speech, spatial orientation and memory, and decreased stability in their emotional state. As the disease mainly affects elderly people, there is also a high risk for other underlying health conditions to be neglected or affected adversely by this disease [1], ultimately increasing their risk of injury or death. In addition, due to the neurodegenerative nature of the disease, steady progression can lead to complications in overall brain functionality. As a result, the typical life expectancy for most patients ranges between 3 and 9 years after their initial diagnosis [6].

As discussed in the next section, AD neuropathology involves the buildup of neuritic plaques of amyloid-β (Aβ) aggregates outside of neurons [7], neurofibrillary tangles of hyperphosphorylated tau, a microtubule-associated protein, within the affected neurons [8] and neuronal loss [9,10]. Therefore, AD pathology and progression have been analyzed by classic postmortem studies, as for example, the Braak staging [11,12,13]. However, recent efforts have focused on developing alternative methods to examine AD neuropathology with the goal to identify initial AD signs antemortem, as for example, using positron-emission tomography (PET) scans for tau and Aβ imaging as discussed below, to indicate that the presence of sufficient quantities of both would permit a diagnosis of AD. 

Based on the progression of mental and intellectual decline, AD is classified into three stages: “Early”, “Middle” and “Late”, as described by the Alzheimer’s Association (www.alz.org, accessed on 24 May 2021). These stages are considered rough generalizations and can be further subdivided into five (www.mayoclinic.org, accessed on 24 May 2021) or even seven stages (www.pennmedicine.org, accessed on 24 May 2021). The first signs can be mistaken for old age, leading to a delayed diagnosis [1]. “Early Stage” hallmarks include difficulty learning new facts or forming new memories, reduction in vocabulary, and some minor difficulties with fine motor tasks [14]. Memories from earlier life and implicit memory, such as how to drink from a glass, are typically not affected at this stage [15].

The “Middle Stage” is when patients often begin to lose their ability to live independently [16]. Vocabulary loss increases dramatically, and motor skills and coordination decrease significantly, leading to much-increased risks of falling and subsequent injury [17]. Long-term memory also becomes impacted, which can lead to the patient having a hard time recognizing close family members [17]. This stage is also characterized by psychosis and erratic behavior, as well as a loss of control of bodily functions such as urination [16]. Due to the nature of these symptoms, patients often begin requiring consistent care and monitoring, and many of them move to assisted-living facilities as a result [18].

The final stage, known as the “Late Stage”, is used to classify patients with the most severe symptoms. Due to the harshness of their symptoms, patients in this stage often lose all independence in their day-to-day lives, requiring around-the-clock monitoring and help, even with the most basic activities [19]. Speech and language skills are almost completely lost, and due to decreased mobility, there is significant muscular atrophy, which ultimately leads patients to be confined to their beds [16]. Due to this bedridden state, one of the most common causes of death for Alzheimer’s patients is the infection of pressure ulcers [20].

Although the cognitive tests described above can help with AD diagnosis in terms of probability, growing efforts have focused on elucidating what is called the “preclinical stage” [21]. Identification of an individual in the asymptomatic preclinical state is accomplished by the in vivo evidence of AD-pathology, which includes the existence of anatomical and molecular AD biomarkers [22,23]. The identification of anatomical and molecular markers via structural and molecular imaging represents a promising method to examine the neuropathology of AD antemortem as proposed by the Alzheimer’s Prevention Initiative (API) [24,25]. Studies on AD biomarkers such as cerebrospinal fluid Aβ42 and tau have indicated a long preclinical phase of the disease of several decades before symptom onset [1,26,27]. Another characteristic of AD progression includes the gradual degeneration of neurons in the cerebral cortex, temporal and parietal lobes [28]. As a result, one of the recently utilized methods to diagnose early disease stages involves magnetic resonance imaging (MRI) to measure regional or whole-brain shrinkage between patients and healthy adults [29]. There is also an assortment of radiopharmaceutical agents used specifically with PET scans to help with the diagnosis, including Florbetapir, Flutemetamol and Florbetaben as Aβ tracers [30], as well as the first approved PET tau tracer, Flortaucipir (trademark name: Tauvid) [31]. Fluorodeoxyglucose (FDG) PET measurements of decline in the cerebral metabolic rate of glucose (rCMRgl) have been recognized as an estimate of neuronal hypometabolism [32,33]. Hypometabolism has been previously proposed as a therapeutic target in AD [34] as well as an independent biomarker [35], particularly after considering observations from autopsy studies in the 1980s showing impairments in brain glucose utilization and energy metabolism [36,37,38,39]. Additionally, other techniques such as blood testing can be used to rule out other causes of cognitive impairment, such as syphilis or heavy metal poisoning.

Different methods have been used to model AD and examine its progression, pathology and responses to various treatments. Stem cell-based organoid development and disease modeling have been proposed as promising novel techniques to investigate AD pathogenesis [40], but several shortcomings and challenges are worth noting. Aging is one of the largest risk factors in AD development, but stem-cell-derived organoids tend to demonstrate transcriptional profiles similar to that of a prenatal brain as well as immature electrical activity patterns, rather than the more complex profiles seen in older individuals [41,42]. Additionally, organoids lack the vascularization observed in brains, and such vascularization is critical in being able to replicate not only the disease, but also the brain anatomy that affects the disease progression [41]. Another challenge is the lack of complexity and diversity of cell types, including reduced numbers of microglia and astrocytes [42]. Finally, there are limitations with regard to the integration of specialized cells such as oligodendrocytes or microglia, or the development of neuronal circuitry that would be of similar complexity to those seen in animal models [41]. Consequently, while the use of stem-cell-derived organoids is an intriguing prospect and there is still much room for improvement, animal models remain a viable approach for replicating the disease pathology, progression and environment.

## 3. AD Pathogenesis and Amyloidogenic APP Processing 

The main neuropathological hallmarks of AD brains include senile plaques and neurofibrillary tangles, as well as neuronal and synaptic loss [43]. Plaques are buildups of processed fragments of the amyloid precursor protein (APP), while tangles are intracellular neurofibrillary buildups of tau proteins [44]. Tau proteins are a group of protein isoforms created through alternative splicing of the microtubule-associated protein tau (MAPT) [45]. They are typically involved in maintaining axon stability through interactions with microtubules, but in AD cases, they become hyperphosphorylated and form neurofibrillary tangles [46]. Although evidence suggests that Aβ deposition and tau pathology can precede neuronal and synaptic loss [47,48,49], especially considering observations from longitudinal imaging of dystrophic neurites and plaques in rodent AD models [50,51,52], the precise timing of the start of neuronal and synaptic loss in AD patients remains to be accurately determined. Despite both plaques and tangles being the main accepted causes of the disease, a recent consensus has been established showing a synergistic effect between the plaques and tangles, and that elimination of the plaques alone can lead to the amelioration of the disease and its symptoms [44,53]. An important factor to consider is the aging-dependent decline in the clearance of plaques from the extracellular space, leading to the Aβ plaque buildup and subsequent development of AD symptoms [54]. Therefore, we will focus our discussion of this review on recent advances in understanding APP molecular roles and interactions.

Amyloid-β (Aβ) is a cleavage product of the Amyloid Precursor Protein, APP [55]. APP is an integral membrane protein, and while it is expressed in a wide variety of different cell types [56], it shows particularly high expression levels on neuronal membranes, especially in synapses [57]. A well-known function of APP surrounds its involvement in the formation and repair of synapses [57]. This APP function is especially evident following neural injury, as well as during the differentiation of neurons when the expression level of APP is significantly upregulated [58]. Additionally, APP is observed to have a trophic function, promoting cell proliferation, differentiation, neurite outgrowth, cell adhesion and synaptogenesis [59] and to be involved in neural stem cell development, neuronal survival and neurorepair [59,60]. Furthermore, APP is also believed to be highly important in reproductive endocrinology, where differential splicing of the protein is key in regulating the differentiation of embryonic stem cells into neural precursor cells [61].

The APP protein is encoded by the gene of the same name, which is located on chromosome 21, spanning 290 kilobases [62]. APP has many different isoforms, and it ranges from 639 to 770 amino acids in length, being the one with 695 amino acids (APP695) the predominant isoform of APP in mammalian neurons [55,63], with a large portion of the protein residing in the extracellular space. This protein is often subjected to a wide range of post-translational modifications, including phosphorylation, glycosylation and proteolytic cleavage [64]. Of these post-translational modifications, proteolytic cleavage seems to be directly involved in the generation of Aβ plaques [65]. There are two cleavage pathways for APP, and they are known as “Amyloidogenic” and “Non-Amyloidogenic” [66]. In the “Non-Amyloidogenic” pathway, the extracellular domain of the APP protein is cleaved by a protease enzyme known as α-secretase. Following the α cleavage, the protein is then cleaved again by γ-secretase, leading to the generation of a soluble larger fragment and a smaller fragment known as p3 [66]. This pathway, as the name implies, does not seem to have any pathogenic effects. However, in the “Amyloidogenic” pathway, rather than being cleaved by α -secretase, the APP extracellular domain is first cleaved by β-secretase (also known as BACE-1), and then by γ-secretase, leading to the generation of another large soluble fragment, but also the Aβ fragment [66]. These Aβ fragments cluster together and become aggregates, forming the aforementioned plaques. 

## 4. APP Genetic and Molecular Interactions

Overexpression of the APP gene significantly increases both AD severity and progression rate, particularly observed in individuals with Down Syndrome (Trisomy 21), who have three copies of this gene and demonstrate AD symptoms as early as 40 years of age [67]. Furthermore, Down Syndrome patients demonstrate a similar buildup of plaques, neurofibrillary tangles, inflammation and oxidative stress as seen in AD patients, and this is believed to be due to triplicate expression of APP [68]. This is further supported by the observation of elevated levels of APP mRNA concentrations of APP in the brains of AD patients [69]. 

To date, a total of 69 mutations in APP have been reported, with32 reported as pathogenic in the Alzforum database (https://www.alzforum.org/mutations/app, accessed on 24 May 2021). One of the most popular APP mutations is known as the “Swedish Mutation”, originally discovered in two separate Swedish families who presented significantly elevated levels of β-amyloid production, along with symptoms characteristic of AD [70,71]. The mutation results in a two amino acid change adjacent to the site of cleavage by BACE-1 on the APP protein, specifically changing lysine and methionine to asparagine and leucine (p.K670N and p.M671L), respectively [72]. This mutation increases the absolute levels of Aβ42 and the rate of protofibril aggregation (without changing the Aβ42 to Aβ40 ratio) [72]. Due to these results, the “Swedish Mutation” has been a popular target for the generation of *Drosophila* and mouse models of Alzheimer’s disease [73].

While the vast majority of mutations discovered on the APP gene are considered to be generally pathological, one recently discovered mutation is believed to be the first to demonstrate a correlative protective effect. The “Icelandic A673T mutation”, as implied by its name, was first found in populations of Iceland and Scandinavia. People heterozygous for this mutation did not have any adverse neurological conditions. On the contrary, they were found to be protected against declines in cognition associated with age [74]. One intriguing report involved a 104-year-old woman heterozygous for the mutation who had little to no amyloid pathology, despite her age and the presence of hippocampal sclerosis [75]. Other reports have demonstrated that individuals with Scandinavian ancestry have similar resilience against Aβ plaque formation and the accompanying pathogenic neurodegeneration [76].

Biologically, the A673T mutation is believed to be similar to the pathogenic Swedish mutation, in that it modifies residues in very close proximity to the primary cleavage site targeted by β-secretase; however, the resulting phenotypes are very different [74]. The “A” residue is the second residue in the β-amyloid domain of the APP protein. This alanine to threonine mutation has several effects. First, it is believed that this mutation results in a less-favorable conformation of the protein for β-secretase to cleave [74]. Second, due to the decreases in cleavage, this mutation also results in reduced Aβ levels [77]. Finally, it is believed that the Aβ fragments which are formed despite the presence of this mutation do not have the same ability to form clusters and show lower Aβ oligomer-binding affinity compared to wild-type Aβ, resulting in almost no aggregation or plaque formation [77,78,79]. Such trends have been observed in mouse and rat models of the A673T mutation, as well as isogenic human-induced pluripotent stem-cell-derived neurons, with the mutation being correlated with decreased amyloidogenic processing of APP, as well as reduction of Aβ aggregation [77,80]. 

APP is an evolutionarily conserved protein, and it is expressed in many different organisms, including *Drosophila* and mice [81,82]. However, it is absent in animals that lack muscles and a nervous system, such as *Trichoplax adhaerens* [83,84]. Although animal nervous systems may lack some complexity and high cognitive functions present in human brains and findings in animal modes may not always translate into efficacious treatments for human patients, the high degree of conservation of fundamental processes animal models supports their use to unravel mechanisms underlying distinct abnormalities and pathophysiological development as well as to develop effective treatment strategies [85]. For example, many models of neurodegenerative diseases have been developed using *Drosophila*, including those for Parkinson’s disease, amyotrophic lateral sclerosis (ALS), Huntington’s disease, Rett syndrome, ataxia telangiectasia and Alzheimer’s disease [86,87,88]. We will focus our discussion on some recent findings from *Drosophila* studies in the section below and highlight some of the novel nutraceutical and synbiotic approaches that have demonstrated promising results in ameliorating APP-dependent phenotypes.

## 5. Recent Research on APP: Insights from *Drosophila*

*Drosophila* expresses the “APP-like” (APPL) protein, which has high homology with human APP (hAPP) in both the N-terminal extracellular domains, as well as the C-terminal intracellular domain [89,90,91]. It is important to note that there are different views about the conservation degree of the domain encoding the Aβ region between both proteins. Whereas some groups indicate that it is not present in *Drosophila* APPL [90,92], other labs have shown that the secreted Aβ-like peptide resulting from APPL cleavage is conserved to hAPP [93,94]. Interestingly, hAPP expression rescues some of the phenotypes observed in *appl* null flies [95], suggesting that functionally, the two proteins are very similar. Conserved motifs between hAPP and *Drosophila* APPL have also been shown to serve the same physiological roles, and that they are sufficient and interchangeable for proper neural functionality [58]. 

Overexpression of wild-type and mutated hAPP has been the strategy of established *Drosophila* AD models for several years (reviewed in [96,97]. Recent studies showed that APP overexpression in flies disrupted sleep patterns, one of the earliest symptoms observed in AD patients [98]. Interestingly, middle-aged flies expressing hAPP demonstrated significant disruption in their sleep patterns, with decreases in daytime and total sleep amounts. This effect was exaggerated in older flies, which revealed consistently increased numbers of sleep bouts and disruption, decreased overall sleep amounts and significant sleep fragmentation [98]. In fact, sleep disorders appear at early AD stages and rise with the severity and progression of AD [99], but the direct mechanisms behind this finding require further investigation. One hypothesis is that regular sleep–wake cycles cause a fluctuation in the amount of Aβ deposition, whereas the development of unclearable plaques disrupts this fluctuation [100]. This results in a positive feedback loop, where reduced sleep leads to reduced Aβ clearance, leading to a further reduction of sleep and a further buildup of plaques [100]. Therefore, powerful genetic tools and a large body of literature on sleep and sleep disorders [101,102,103] make *Drosophila* a promising model to further investigate the relationship between sleep, APP and human disorders.

A recent study in *Drosophila* demonstrated disruption of autophagy following the altered expression of the activating subunit of the Cdk5 protein kinase (Cdk5α) [104]. Such disruption of autophagy caused the hyperactivation of innate immunity, which in turn induced the age-dependent death of dopaminergic neurons, establishing a fly model to study autophagy, innate immunity and neurodegeneration [104,105]. The autophagic pathway and innate immunity have been involved in neurodegenerative diseases, including AD [106]. In the context of AD, previous research has not clarified whether this dysregulation is a cause or effect of the pathological state. Research by Zhuang and colleagues demonstrated that the aberrant autophagy seen in *Drosophila* AD models is due to abnormally elevated levels of APP, which leads to a positive feedback loop of dysregulation in APP metabolism and further worsened symptoms. The protein chaperone E3 ligase known as CHIP (carboxyl-terminus of Hsc70-interacting protein), which is a key component in the autophagic pathway, induces Aβ production by increasing the expression of BACE-1, leading to aberrant autophagy and subsequent neurodegeneration [107]. A suppression of APP-induced neurodegenerative effects in eye development was observed by downregulating CHIP activity [107]. Additional APP-induced deficits in wing expansion, locomotion and an overall reduction in lifespan were all ameliorated by the depletion of the CHIP chaperone E3 ligase [107]. Human CHIP shares ∼60% amino acid sequence similarity with fly CHIP [108], and is involved in high metabolic activity and protein turnover, but no role of CHIP in APP regulation and AD pathogenesis was known.

Another *Drosophila* study examined the role of APP in memory formation and memory loss, as one of the keystone symptoms of AD is a loss of both short and long-term memory. Using RNA interference, interactions between the intracellular domains of APPL and membrane-associated guanylate kinase proteins (MAGUK) were shown to be critical for appetitive long-term memory, memory which is needed for intrinsic survival functions such as eating and drinking [109]. Additionally, their genetic analysis suggested that these interactions would not only be present in *Drosophila*, but may be conserved across many different species, including humans [109]. Consistently, deficits in learning and memory have been previously reported not only in appl null flies [110,111], but also in global APP knockout mice [112]. In fact, some neurons in the learning and memory center of the fly brain, the mushroom bodies, presented modestly penetrant axonal defects in *appl* null flies [113], a process that is thought to involve interactions between APPL, the Wnt-PCP signaling pathway, the tyrosine kinase Abl and the fly huntingtin protein Htt [114]. Additional phenotypes recently described in *appl* null flies include a significant compromise in survival at early ages, neuronal cell death, enlargement of early endosomal compartments and the accumulation of dead neurons in their brains [115]. Consistently, global APP knockout mice have also demonstrated significant impairments in cerebral blood flow, especially when exposed to hypoxic conditions, ultimately causing acute mortality [116]. 

## 6. The Use of Nutraceuticals as Promising Treatment Options

γ-secretase was the target of one of the first drugs (Semagacestat) meant to treat AD. It unfortunately had very dire consequences and was canceled during phase 3 clinical trials over safety concerns (e.g., patients treated with Semagacestat had a significantly higher incidence of skin cancer than those who were given a placebo) and worsening of the pathological conditions [117]. Whether these results were due to the targeting γ-secretase has never been determined and lends support to the need to understand the basic biology of these proteins. Similarly, since BACE-1 is a unique component of the Amyloidogenic pathway, there have been quite a few BACE-1 inhibitor drugs undergoing clinical trials [65]. However, adverse effects have been observed in many drug trials targeting BACE-1. For instance, “Verbucestat”, a novel BACE-1 inhibitor from Merck, was shown to cause increases in falls and injuries, suicidal thoughts, sleep disturbance and other undesirable side effects [118]. Janssen also suspended their BACE-1 inhibitor “Atabecestat” during their phase 2 clinical trials due to liver toxicity [119]. Other attempts at inhibiting BACE-1 have brought about impairment of synaptic transmission, plasticity and long-term hippocampal potentiation, which ultimately bring into question whether or not this therapeutic treatment will be effective and non-invasive [65]. A comprehensive description of some of the drugs described above, as well as additional promising therapeutic and synthetic agents, has been recently reviewed [120,121,122]. 

A more promising approach to reduce BACE-1 activity has been recently shown in *Drosophila* AD models, which involves a nutraceutical treatment with gallic acid [123]. Gallic acid is a trihydroxybenzoic acid found in a wide variety of plants, ranging from sumac to tea leaves, oak trees and blue-green algae. It is classified as a phenolic acid with strong antioxidant and free radical scavenging properties. It is also found in many edible fruits, such as strawberries, bananas and grapes. AD-flies exposed to different concentrations of gallic acid in their diet (50 and 100 µM) showed reduced activity of cholinesterases and β-secretase (BACE-1) as well as concentrations of reactive oxygen species and malondialdehyde [123]. The therapeutic potential of gallic acid is exciting, as elevated BACE-1 activity is often seen in severe AD cases and leads to increases in Aβ production. While previous efforts to inhibit BACE-1 demonstrated undesirable side effects, it is possible that reduction of BACE-1 activity in conjunction with a reduced level of oxidative stress is achievable by administering gallic acid, and this can offer a worthwhile therapeutic pathway [123,124]. In a mouse model of AD, gallic acid also reduced β-secretase activity, inhibited neuroinflammation and stabilized brain oxidative stress [125], further supporting the observations using the fly model. Other studies indicated that administration of gallic acid caused reductions in neuronal reactive oxygen species, improvement of learning and memory and improved brain electrical activity [126]. 

Exposure to extraction of Mulberry fruit has also shown beneficial results in reducing Aβ toxicity in *Drosophila* AD models [127]. In extracts from mulberry fruit of *Morus* cf. *nigra* “Chiang Mai” obtained using acidic methanol, the only anthocyanin detected by the authors was cyanidin, with a content of around 250 ɥg/g dry weight [127]. The principal component of this extract is anthocyanin, which is a flavonoid that is naturally found in the tissues of many higher plants. Anthocyanin, similar to gallic acid, has been shown to have antioxidant properties, as well as benefits in the gastrointestinal system [128]. The study conducted by Suttisansanee showed the extract inhibiting BACE-1 and cholinesterase activity, in addition to promoting neurite outgrowth in the neuronal cells [127]. Additionally, previous studies in mice have shown links between the administration of anthocyanin and improvement in Aβ clearance, reduction of inflammation and halting of neurodegeneration [129]. Furthermore, other studies have shown that anthocyanin is able to work synergistically with gallic acid in modulating BACE-1 activity, reducing inflammation and improving the clearance of Aβ plaques [130]. Therefore, a combination therapy of gallic acid and anthocyanin may prove to be worth exploring to examine whether it is able to provide therapeutic benefits to AD patients, as both compounds are known to cross the blood–brain barrier [131,132]. It is worth emphasizing that whereas the studies mentioned above suggest promising and correlative results between the use of nutraceuticals and the amelioration of some AD-related phenotypes, further studies are required to confirm that the doses of nutraceuticals given to the animals are relevant to the observed biological effects.

Although the focus of this review is on recent publications using *Drosophila* AD models, the studies described above represent a small fraction of nutraceutical studies not only in flies, but, even in a larger number, in rodent models [122,133,134]. For example, cinnamon and its active compound cinnamaldehyde have also shown beneficial effects on fly and mouse AD models [135,136,137]. Some additional nutraceuticals that have shown promising results ameliorating AD-related phenotypes (Table 1) include the flavonoid silybin B [138,139], curcumin [140], saffron [141], sulforaphane [142], iron [143,144] and other transition metals [145]. In fact, silver treatment in flies [146] represents an intriguing treatment option that requires further investigation as silver is known to have opposite effects, including antiseptic activity and reduced brain inflammation as well as neurotoxicity [147,148]. 

When discussing nutraceutical approaches and AD, it is important to include the gut–brain axis and the emerging evidence of multiple interactions between gut dysbiosis and AD [149,150]. Interestingly, a recent study shows that flies raised without a bacterial microbiome failed to show the age-related increase in activation of the immune response genes and decline in expression of stress response genes observed in control flies [151]. These results indicate a crucial role of the gut microbiome in aging, since age-dependent systemic changes in gene expression, particularly stress and immune response genes, fail to happen when flies are grown axenically [151]. In murine AD models, previous literature reports that the gut microbiome can influence the neuroinflammation in AD through the production of proinflammatory cytokines (IL-1*β*, IL-6, IL-18, TNF-*α* and IFN-*γ*) and bacterial metabolites [152]. *Drosophila* AD models show increased levels of the TNF-*α* *eiger*, whose downstream activator JNK causes inflammation-induced apoptosis [153]. Enteric dysbiosis by oral infection with non-pathogenic enterobacteria was induced in AD flies, which strongly exacerbated neurodegeneration via immune hemocyte recruitment to the brain. These results suggest that the gut–brain axis promotes neurodegeneration by the mobilization of hemocytes and their attraction to the diseased brain [153]. Furthermore, promising results including improvement of gut dysbiosis, altered microbiota-derived metabolites, neuroinflammation and cognition impairment were observed in AD transgenic mice and in initial clinical trials in humans after treatment with sodium oligomannate (GV-971), a mixture of oligosaccharides derived from marine brown algae [154,155]. It would be interesting to test GV-971 in *Drosophila* AD models to further investigate its underlying molecular roles and effects in neuronal anatomy, such as spine remodeling [121] and synaptic refinement [156].

The use of probiotics has been described as an efficient strategy in the treatment of various neurological conditions [157]. A reduction in the onset and progression of disease-related phenotypes was recently described by the modulation of the gut–brain axis through probiotic treatment in AD flies [158]. A synbiotic formulation containing three bioactive probiotics (*Lactobacillus plantarum* NCIMB 8826 (Lp8826), *L. fermentum* NCIMB 5221 (Lf5221), and *Bifidobacteria longum* spp. infantis NCIMB 702255 (Bi702255)) and a novel polyphenol-rich prebiotic, Triphala (TFLA), improved survival, motility, Aβ accumulation and acetylcholinesterase activity, likely acting through mechanisms implicating the peroxisome proliferator-activated receptor (PPAR)γ [158]. In a mouse AD model, Bonfili et al. (2020) [159] investigated the effects of probiotics (SLAB51) in restoring glucose homeostasis. Glucose uptake correlates with a higher risk of developing AD and is influenced by abnormalities of AMPK and Akt. Results showed restored expression levels of glucose transporters (GLUT1 and GLUT3) and reduced phosphorylation of tau, AMPK and Akt after treatment of SLAB51 in AD mice. Moreover, SLAB51 counteracted insulin resistance and improved glucose metabolism impairment, delaying AD progression. In humans, Nagpal et al. [160] compared the effects of dietary intervention on the microbiome of normal versus mild cognitively impaired (MCI) subjects. Gut microbial signatures such as reduced SCFAs and a greater abundance of proinflammatory bacteria were present in MCI subjects. Results showed that a modified Mediterranean-ketogenic diet (MMKD) regulated the gut microbiome and the production of its metabolites, improving AD symptoms. Although there are several factors that limit these studies, such as small sample size and gender bias, the findings provide relevant information on the role of the gut microbiota–brain axis in AD and contribute to the development of therapeutics.

## 7. Concluding Thoughts

Alzheimer’s disease has been and will continue to be a detrimental burden on the elderly population. Not only does it lead to a significant reduction in the quality of life for both patients and their families, but it also has the potential to distress the healthcare industry. Model organisms such as *Drosophila* have been utilized to investigate relevant molecular tools and examine subsequent phenotypes, broadening our understanding of fundamental mechanisms. In fact, besides the results described above, additional genetic screens and molecular studies in *Drosophila* have expanded our understanding of additional AD-associated loci [161].

**Figure 1:**
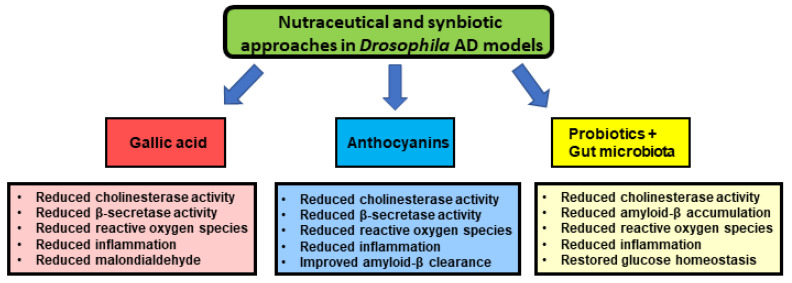
Diagram of recent nutraceutical and synbiotic approaches tested in *Drosophila* AD models and their molecular and cellular effects. Future studies would confirm that the doses of nutraceuticals given to the flies are relevant to the biological effects observed in the initial studies discussed in the text.

Furthermore, whereas our discussion focused on the success of nutraceutical approaches (Figure 1), conclusive evidence on their validity in the treatment of human AD from clinical studies is still lacking. Besides nutraceuticals, a few treatments are worth noting based on their promising initial results in animal models, including pharmacological treatments such as histone deacetylases inhibitors [162] and angiotensin-converting enzyme inhibitors [163,164,165]. Additionally, whereas evidence from current clinical trials indicates beneficial effects of acetylcholinesterase inhibitors [4] and anticancer drugs in AD patients [166], the future application of genetic approaches such as gene therapy [167], including RNA-based therapy [168] and CRISPR [169] represents a new window for AD treatment. Additionally, the growing literature indicating a role of the gut microbiome in host physiology, metabolism, disease-associated phenotypes and efficacy of drug therapies underscores the relevance of this evolving research field. Despite differences between the more complex mammalian microbiome and the relatively simpler fly one, several advantages of the *Drosophila* gut microbiota have been reported, supporting its use as a model to study mammalian gut complexity and microbiome/drug interactions [170,171]. Future studies will determine its relevance in expanding our understanding of fundamental mechanisms underlying neurological diseases. 

By contrast, it may be worth considering the role of developmental processes in AD onset and progression. This idea is supported by the observations that not only mammalian APP and *Drosophila* APPL play important roles in developmental processes as described above, but also other AD-associated genes and proteins are involved in neurodevelopmental processes such as neuronal migration and axon extension [172]. Finally, recent advances in molecular biology have allowed the emergence of a large body of exciting evidence linking AD-related mechanisms and epigenetics [173,174]. Several fundamental epigenetic mechanisms are conserved in flies, supporting the idea that the insight gained from *Drosophila* studies will likely advance the understanding of the mammalian brain, and thus be relevant to human health.

## Figures and Tables

**Table 1:** Nutraceutical compounds known to ameliorate phenotypes in animal AD models.

Compound	Preferred IUPAC name	Chemical Formula	Type of Molecule	Occurrence	References (AD Models)
Gallic acid	3,4,5-Trihydroxybenzoic acid	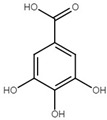	Phenolic acid	Sumac (*Rhus*), tea leaves, strawberry, grape	[123,124,125]
Cyanidin	2-(3,4-Dihydroxyphenyl)-3,5,7-trihydroxy-1λ4-benzopyran-1-ylium	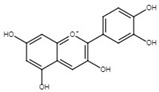	Pigment	Mulberry (*Morus nigra)* Blueberry *(Vaccinium*)	[127,129,130]
Cinnamaldehyde	(2E)-3-Phenylprop-2-enal	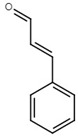	Phenylpropanoid	Cinnamon	[135,136,137]
Curcumin	(1E,6E)-1,7-Bis(4-hydroxy-3-methoxyphenyl)hepta-1,6-diene-3,5-dione	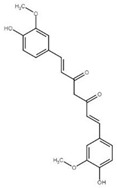	Diarylheptanoid	Turmeric (*Curcuma longa*)	[140]
Silybin	(2*R*,3*R*)-3,5,7-trihydroxy-2-[(2R*,3R*)-3-(4-hydroxy-3-methoxyphenyl)-2-(hydroxymethyl)-2,3-dihydrobenzo[*b*][1,4]dioxin-6-yl]chroman-4-one	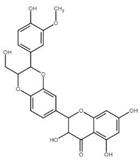	Flavonolignan	Milk thistle (*Silibum marianum*)	[138,139]
Crocin	Bis[(2*S*,3*R*,4*S*,5*S*,6*R*)-3,4,5-trihydroxy-6-({[(2*R*,3*R*,4*S*,5*S*,6*R*)-3,4,5-trihydroxy-6-(hydroxymethyl)oxan-2-yl]oxy}methyl)oxan-2-yl] (2*E*,4*E*,6*E*,8*E*,10*E*,12*E*,14*E*)-2,6,11,15-tetramethylhexadeca-2,4,6,8,10,12,14-heptaenedioate	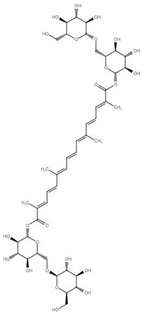	Carotenoid	Saffron	[141]
Sulforaphane	1-Isothiocyanato-4-(methanesulfinyl) butane	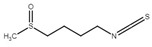	Isothiocyanate	Broccoli Brussel sprouts Cabbage	[142]
